# Cephalotaxine Inhibits the Survival of Leukemia Cells by Activating Mitochondrial Apoptosis Pathway and Inhibiting Autophagy Flow

**DOI:** 10.3390/molecules26102996

**Published:** 2021-05-18

**Authors:** Tingting Liu, Qiang Guo, Shuze Zheng, Yang Liu, Heng Yang, Meimei Zhao, Lu Yao, Kewu Zeng, Pengfei Tu

**Affiliations:** 1 State Key Laboratory of Natural Medicines, China Pharmaceutical University, Nanjing 210009, China; cpultt126@126.com; 2 State Key Laboratory of Natural and Biomimetic Drugs, School of Pharmaceutical Sciences, Peking University, Beijing 100191, China; qiangguo@pku.edu.cn (Q.G.); 1610307325@pku.edu.cn (S.Z.); 1711110083@pku.edu.cn (Y.L.); upyangheng@163.com (H.Y.); meimeizhao@pku.edu.cn (M.Z.); yaoolu@stu.pku.edu.cn (L.Y.)

**Keywords:** cephalotaxine, leukemia cells, RNA-sequencing, mitochondrial apoptosis pathway, autophagy flow

## Abstract

Cephalotaxine (CET) is a natural alkaloid with potent antileukemia effects. However, its underlying molecular mechanism has not been well understood. In this study, we verified that CET significantly inhibited the viability of various leukemia cells, including HL-60, NB4, Jurkat, K562, Raji and MOLT-4. RNA-sequencing and bioinformatics analysis revealed that CET causes mitochondrial function change. Mechanism research indicated that CET activated the mitochondrial apoptosis pathway by reducing the mitochondrial membrane potential, downregulating anti-apoptotic Bcl-2 protein and upregulating pro-apoptotic Bak protein. In addition, the autophagy signaling pathway was highly enriched by RNA-seq analysis. Then, we found that CET blocked the fluorescence colocation of MitoTracker Green and LysoTracker Red and upregulated the level of LC3-II and p62, which indicated that autophagy flow was impaired. Further results demonstrated that CET could impair lysosomal acidification and block autophagy flow. Finally, inhibiting autophagy flow could aggravate apoptosis of HL-60 cells induced by CET. In summary, this study demonstrated that CET exerted antileukemia effects through activation of the mitochondria-dependent pathway and by impairing autophagy flow. Our research provides new insights into the molecular mechanisms of CET in the treatment of leukemia.

## 1. Introduction

Leukemia is a disease of hematopoietic malignancy, which is a common cancer type for adults and children. Leukemia can be divided into chronic and acute varieties, both of which include lymphocytic and myelocytic types [1]. Cytogenetic and molecular genetic abnormalities play a vital role in hematological malignancies. The abnormalities of leukemia mainly include gene rearrangements and mutations, activation of proto-carcinogenic genes, inactivation of tumor suppressor genes, etc. Specific gene changes have become important molecular markers for the diagnosis and treatment of leukemia. Gene mutations in leukemia cause the malignant proliferation of leukemia cells and increase their resistance to chemotherapeutic drugs. Therefore, the detection of chromosomes and gene mutations is helpful to the diagnosis and prognosis of leukemia [2,3,4]. At present, therapeutic strategies for leukemia include radiotherapy, chemotherapy and bone marrow transplantation. Chemotherapy is an important method for the therapy of leukemia. However, it shows severe side effects, including toxicity and drug resistance. Thus, developing novel and reliable therapeutic strategies seems to be an urgent need.

Autophagy plays an important role for the maintenance of cellular homeostasis. Autophagy is a self-eating and lysosomal-dependent degradation process, which is involved in the clearance of damaged organelles and misfolded proteins [5]. In general, autophagy acts as a cytoprotective role in hematological cancers, including acute myeloid leukemia (AML), chronic lymphocytic leukemia (CLL) and chronic myeloid leukemia (CML) [6,7]. Accordingly, inhibition of autophagy contributes to the treatment of leukemia. For example, inactivation of autophagy through knocking out Atg5 or Atg7 could prolong the survival of leukemic mice in AML model [8]. The miR-130a could mediate the downregulation of *ATG2B* and *Dicer1* genes to inhibit autophagic flux in CLL cells, and drive CLL cell death [9]. In addition, autophagy is necessary to maintain the function of mitochondria. Inactivation of autophagy leads to the accumulation of morphologically abnormal mitochondria in tumors. Damaged mitochondria cannot be effectively eliminated in autophagy-deficient cancer cell lines [10]. Based on this information, it can be assumed that blocking the autophagy pathway may promote mitochondria-dependent apoptosis in cancer cells. For example, chemotherapy drugs, such as bortezomib, typically activate the mitochondrial apoptotic pathway and result in leukemia cells’ death. Inhibition of autophagy could enhance the antileukemia activity of bortezomib [11]. Leukemia cells themselves can produce protective autophagy against adverse environmental stimuli, which leads to cell resistance to chemotherapy drugs [12]. Therefore, targeting the autophagy process may serve as a good therapeutic strategy for leukemia, and it is also essential to develop new autophagy regulators.

Cephalotaxine (CET) is an alkaloid isolated from the Chinese coniferous tree *Cephalotaxus hainanensis* or *Cephalotaxus drupacea* [13]. CET has effective antiviral activities against Zika and hepatitis B virus [14,15] and also shows promising antileukemia activity [16]. It has been reported that CET exerts antileukemia activity by promoting cell apoptosis. However, the underlying mechanism remains poorly understood. In the present study, it was firstly demonstrated that CET induced apoptosis of leukemia cells by activating the mitochondrial apoptosis pathway. In addition, CET inhibited lysosomal acidification and interfered with autophagy flux. Inhibition of autophagic flux interfered with the removal of damaged mitochondria and aggravated cell apoptosis. Collectively, our research provides a new therapeutic strategy for leukemia.

## 2. Results

### 2.1. CET Induces Apoptosis of Leukemia Cells

**Figure 1:**
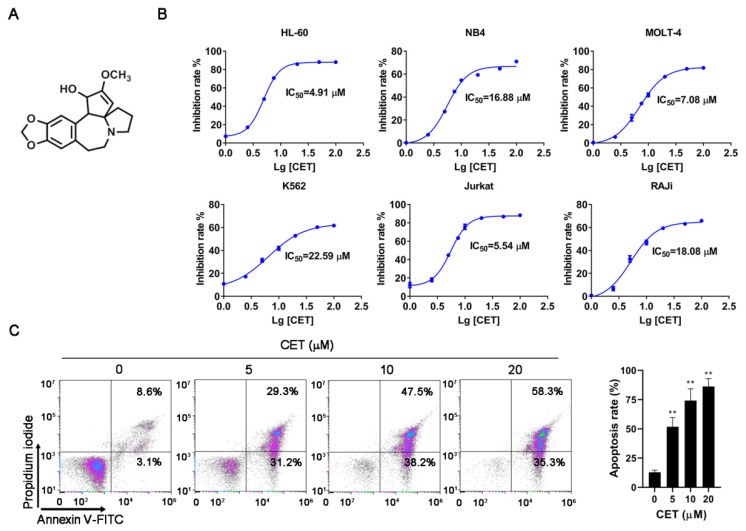
CET induces cell apoptosis in various leukemia cells. (**A**) Chemical structure of CET. (**B**) Cells were treated with CET for 24 h. Effects of CET on the cell viability of different leukemic cell lines were determined by CCK-8 assay. (**C**) HL-60 cells were incubated with CET (5, 10, 20 μM) for 24 h, stained by annexin V-FITC/PI and analyzed by flow cytometry. Data are representative of three experiments. ** *p* < 0.01 versus control group.

The effects of CET on various leukemia cells were detected by a CCK-8 assay. The leukemia cells, including HL-60, NB4, MoLT-4, Jurkat, K562 and Raji, were exposed to different concentrations of CET (1–100 μM) for 24 h. The results revealed that CET had differential cytotoxic effects on these cells. The IC_50_ values of CET for HL-60, NB4, MoLT-4, K562, Jurkat and Raji cells were about 4.91, 16.88, 7.08, 22.59, 5.54 and 18.08 μM, respectively. Moreover, CET treatment exhibited stronger cytotoxicity in both HL-60 cells and Jurkat cells (Figure 1B). Thus, HL-60 cells were selected for subsequent experiments. HL-60 cells were treated with CET (0, 5, 10, 20 μM) for 24 h, and cell apoptosis was measured by flow cytometry with annexin V-FITC/PI staining. The results showed that the apoptosis rate of HL-60 cells was significantly increased, compared with that of the control group (Figure 1C). Thus, CET shows antileukemia effects by inducing cells apoptosis in vitro.

### 2.2. Transcriptome Sequencing-Coupled with Bioinformatics Analysis Reveals Signaling Networks Regulated by CET

**Figure 2:**
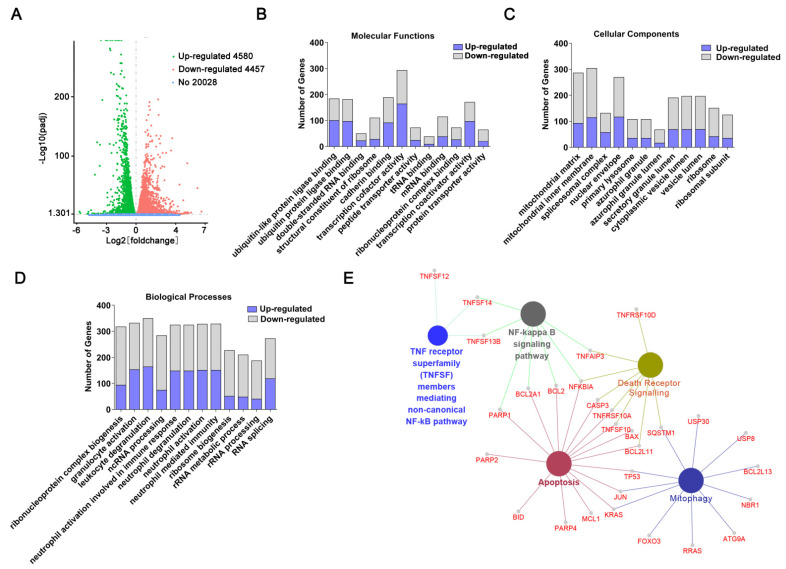
Transcriptome analysis of CET-altered genes. Cells were treated with vehicle (control group) or CET (20 µM) for 12 h, respectively. (**A**) The volcano plot represents differentially expressed genes. Gene Ontology enrichment analysis of differential genes included molecular functions (**B**), cellular components (**C**) and biological processes (**D**). (**E**) KEGG pathway enrichment analysis was determined using the ClueGO plug-in.

To investigate the CET-regulated signaling pathway, transcriptome sequencing was conducted in this study. The volcano plot could clearly show differential gene distribution. As shown in Figure 2A, 4580 genes were upregulated, while 4457 genes were downregulated after CET treatment. To further reveal the potential functions of differentially expressed genes, GO enrichment analysis was carried out. The analysis mainly focused on molecular functions (MFs), cellular components (CCs) and biological processes (BPs). The results showed that differentially expressed genes had diverse activities, mainly including transcription cofactor activity, cadherin binding and ubiquitin-like protein ligase binding (Figure 2B). These genes were mostly located in the mitochondrial matrix and mitochondrial inner membrane (Figure 2C). According to BPs’ classification, the differential genes were involved in diverse biological processes, containing ribonucleoprotein complex biogenesis, neutrophil activation in immune response, and so on (Figure 2D). Therefore, based on GO analysis, CET induced changes in protein transcription, translation and mitochondrial function. In addition, KEGG enrichment analysis was determined using the ClueGO plug-in. Some major pathways were enriched, including apoptosis, autophagy, death receptor signaling and NF-κB signaling pathway (Figure 2E). Based on previous studies, mitophagy is also involved in the caspase-mediated cell death process and acts as a therapeutic target to reduce cancer progression by inducing mitochondrial dysfunction [17,18]. Therefore, we speculate that CET may induce mitochondrial function changes and regulate the autophagy pathway.

### 2.3. CET Activates the Mitochondria-Dependent Pathway in Leukemia Cells

**Figure 3:**
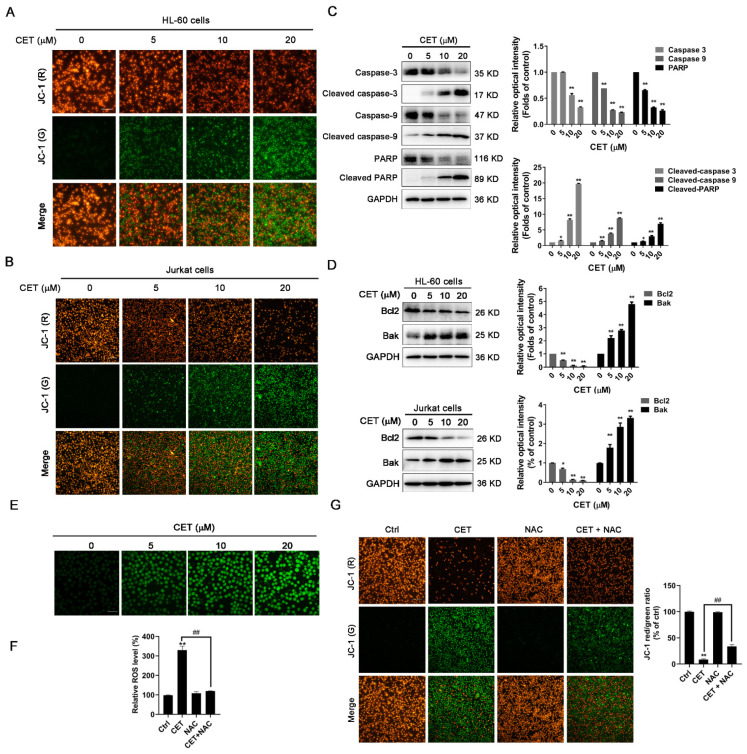
CET induces cell apoptosis via the mitochondria-dependent pathway in leukemia cells. (**A**) JC-1 staining was performed to investigate mitochondrial membrane potential of HL-60 cells treated with CET treatment (5, 10, 20 µM) for 24 h. The cells with depolarized mitochondria were identified by green fluorescence, scale bar = 50 µm. (**B**) JC-1 staining was performed to investigate the mitochondrial membrane potential of Jurkat cells treated with CET treatment (5, 10, 20 µM) for 24 h. (**C**) The expression levels of PARP, caspase-9, caspase-3 and their cleaved forms in HL-60 cells were examined by Western blot analysis. The band intensities were determined by Image J software and normalized against the GAPDH signal. (**D**) The protein levels of Bcl-2 and Bak in HL-60 and Jurkat cells were detected by Western blot. (**E**) HL-60 cells were treated with CET treatment (5, 10, 20 µM) for 24 h. The intracellular ROS production was assessed by H2DCFDA staining assay. (**F**) HL-60 cells were treated with 20 μM CET alone or in combination with NAC (5 mM) for 24 h to measure ROS levels. (**G**) The changes in mitochondrial membrane potential were assessed by JC-1 staining in HL-60 cells treated with 20 μM CET alone or in combination with NAC (5 mM) for 24 h. All experiments were performed in triplicate. Data are expressed as mean ± SD (n = 3). * *p* < 0.05 and ** *p* < 0.01 versus control group. ^##^ *p* < 0.01 versus CET group.

To investigate the effects of CET on mitochondrial functions, mitochondrial membrane potential (Δψm) was measured with a potential-sensitive dye, JC-1. In healthy cells, JC-1 forms J-aggregates at high values of Δψm and emits red fluorescence in the mitochondria. While, in the cells with depolarized mitochondria, JC-1 exists as a monomer at low values of Δψm and emits green fluorescence [19]. As shown in Figure 3A,B, the green fluorescence intensity was significantly increased after CET treatment, while the red fluorescence intensity was remarkably decreased. The results indicated that CET could notably decrease the mitochondrial membrane potential of HL-60 and Jurkat cells in a concentration-dependent manner. Additionally, CET significantly increased the expression levels of cleaved caspase-3, caspase-9 and PARP, indicating the activation of the caspase-dependent apoptotic pathway (Figure 3C). To further explore the expression of apoptosis-associated proteins involved in the mitochondrial pathway, the levels of Bcl-2 and Bak were assessed by Western blot analysis. The results demonstrated that CET upregulated the expression of pro-apoptotic protein Bak and correspondingly downregulated the expression of anti-apoptotic proteins Bcl-2 in HL-60 and Jurkat cells in a concentration-dependent (Figure 3D) and time-dependent manner (Figure S1, In the Supplementary Materials). Meanwhile, the amount of reactive oxygen species (ROS), a byproduct of mitochondrial damage, was determined by H2DCFDA staining. Our data showed that CET-induced mitochondrial damage resulted in the accumulation of intracellular ROS (Figure 3E). Next, we used NAC, a ROS inhibitor, to examine whether the increasing ROS in turn caused the mitochondrial dysfunction. As expected, NAC strongly inhibited the generation of ROS induced by CET (Figure 3F). However, NAC partly reversed the CET-induced decrease in Δψm (Figure 3G). Therefore, CET-induced mitochondrial damage could be partly due to the accumulation of ROS. Altogether, these results suggested that CET induced the apoptosis of leukemia cells via the mitochondrial-dependent pathway.

### 2.4. CET Interferes with Mitochondrial Homeostasis by Regulating Autophagy

**Figure 4:**
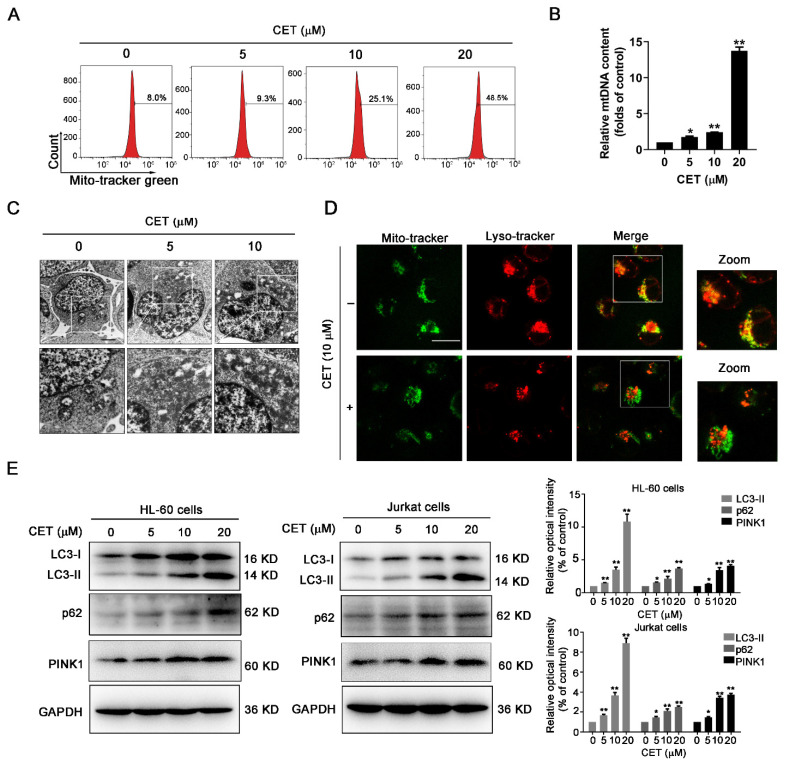
CET regulates autophagy. (**A**) HL-60 cells were treated with CET (5, 10, 20 μM) for 24 h. The fluorescence intensity of cells stained with MitoTracker Green was quantified by flow cytometry. (**B**) The mtDNA content of CET-treated cells was measured by qPCR analysis. (**C**) HL-60 cells were treated with CET (5, 10 μM) for 24 h. Then transmission electron microscopy was used to observe the structure of cells (magnification 12,000×). (**D**) The colocalization of mitochondria and lysosomes was observed in HL-60 cells, which were stained with MitoTracker Green and LysoTracker Red. Images were captured with a confocal laser scanning microscope (scale bar = 10 µm). (**E**) The expression levels of p62, LC3-II and PINK1 in HL-60 and Jurkat cells were determined by Western blot. Data are presented as the mean ± SD. * *p* < 0.05 and ** *p* < 0.01 versus control group.

In this study, HL-60 cells were stained with MitoTracker Green, and the quantification of green fluorescence intensity was carried out using flow cytometry. As shown in Figure 4A, CET induced a significant increase of green fluorescence intensity in a dose-dependent manner. CET might induce the accumulation of damaged mitochondria. To further determine the accumulation of damaged mitochondria in CET-treated cells, the mtDNA content was measured by qPCR analysis. The findings showed that CET treatment caused a significant increase of mtDNA content (Figure 4B). The ultrastructure of HL-60 cells was observed by transmission electron microscopy. The accumulation of damaged mitochondria was also detected in the CET-treated group (Figure 4C). These results demonstrated that CET could induce the accumulation of damaged mitochondria and interfere with mitochondrial homeostasis.

Based on the transcriptome analysis, the mitophagy signaling pathway was enriched in the CET-treated group. Therefore, the effect of CET on autophagy was further investigated. Mitophagy is a major pathway for the elimination of damaged mitochondria [20]. Therefore, we speculated that CET might affect the mitophagy or autophagy. The colocalization of mitochondria and lysosomes was detected by MitoTracker Green and LysoTracker Red staining. The result showed that CET significantly decreased the overlay of mitochondria and lysosomes, suggesting that autophagy (mitophagy) was impeded in the CET-treated group (Figure 4D). LC3 has been used as a typical marker of autophagosome. When autophagy is induced, the cytosolic form of LC3-I is converted to LC3-II via phosphatidylethanolamine conjugation. Then, autophagosomes are assembled by recruiting LC3-II to autophagosomal membranes [21]. Thus, to further determine the effects of CET on autophagy, the expression levels of LC3-II in HL-60 and Jurkat cells were investigated by Western blot analysis. The results showed that CET increased the level of LC3-Ⅱ (a marker of autophagosomes) in a concentration-dependent (Figure 4E) and time-dependent manner (Figure S1). The upregulation of autophagosomes might be caused by the initiation of autophagy or the suppression of autophagic degradation. Based on related experiments, CET increased PINK1 levels in HL-60 and Jurkat cells, indicating that autophagy initiation was induced. If autophagy was induced, why did CET promote the accumulation of mitochondria? We speculated that the downstream of autophagy may be blocked. To further clarify the mechanism, the expression level of p62 in HL-60 and Jurkat cells was also evaluated. Usually, p62 is degraded via the autophagy–lysosome pathway, and the level of p62 is negatively related to autophagy activity. As shown in Figure 4E, CET treatment caused the upregulation of p62 in a dose-dependent fashion, suggesting that CET inhibited the autophagy activity. Therefore, CET effects mitochondrial homeostasis by regulating autophagy.

### 2.5. CET Inhibits Autophagy Flow

**Figure 5:**
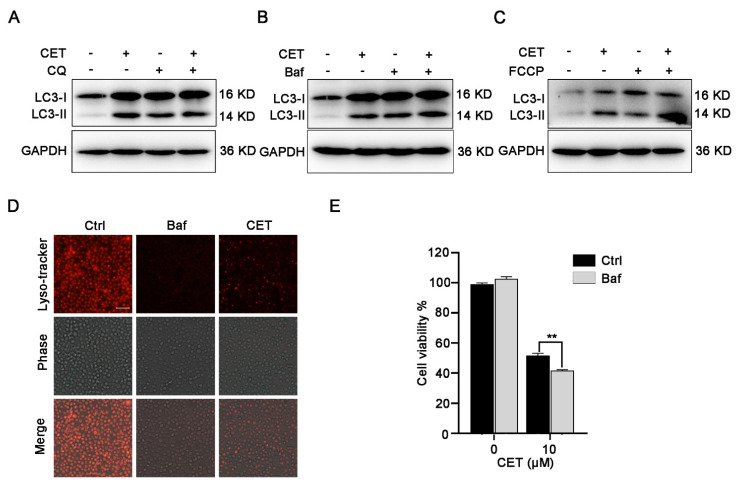
CET suppresses autophagy flux. (**A**) In the absence or existence of CQ (10 µM), HL-60 cells were treated with CET (10 µM) for 24 h. LC3-II was measured by Western blotting. (**B**) In the absence or existence of bafilomycin A1 (Baf, 100 nM), HL-60 cells were treated with CET (10 µM) for 24 h. LC3-II was measured by Western blotting. (**C**) In the absence or existence of FCCP (20 µM), HL-60 cells were treated with CET (10 µM) for 24 h. LC3-II was measured by Western blotting. (**D**) CET inhibited the acidification of lysosomes in HL-60 cells. LysoTracker Red was used as a fluorescent probe of acid lysosomes (scale bar = 50 µm). (**E**) In the presence or absence of Baf (100 nM), cells were treated with 10 µM CET for 24 h. Cell viability was determined by the CCK-8 assay. ** *p* < 0.01.

CET may regulate autophagy by affecting the downstream of autophagy. Therefore, we confirmed this with autophagy inhibitors, chloroquine (CQ) or Baf A1. Both CQ and Baf A1 are potent autophagy inhibitors by blocking of autophagic flux. As shown in Figure 5A,B, treatment with CQ or Baf A1 significantly promoted the accumulation of LC3-II in HL-60 cells. CET in combination with Baf A1 or CQ did not further increase the level of LC3-II compared to that in the cells treated with CET alone, indicating that CET interfered with the degradation of autophagosomes and inhibited autophagy flow. In addition, FCCP, as mitophagy-specific inducer can increase autophagosome synthesis. As shown in Figure 5C, FCCP induced further accumulation of the LC3-II protein in CET-treated cells. The result also revealed that CET inhibited autophagy flow. Meanwhile, similar to Baf A1, a canonical inhibitor of lysosomal acidification, CET also impaired lysosomal acidification (Figure 5D). Therefore, these results indicated that the inhibition of autophagy flux induced by CET was correlated with lysosomal pH alteration. We also found that Baf A1 exacerbated CET-induced cell death (Figure 5E). Thus, these results revealed that CET suppressed the downstream of autophagy, and further inhibition of autophagy flux contributed to cell death induced by CET.

## 3. Discussion

CET exerts an effective antileukemia activity. However, there is no research about the underlying mechanism of its antileukemia effects. The potential molecular mechanisms of CET-induced apoptosis remain to be elucidated. Here, our data demonstrated that CET inhibited leukemia cells’ survival by activating the mitochondrial-dependent apoptosis pathway and interfering with autophagy flow. Inhibition of autophagy flux caused the accumulation of dysfunctional mitochondria, disrupted mitochondrial homeostasis and promoted HL-60 cells apoptosis. Further inhibition of autophagic flux could aggravate cell apoptosis induced by CET. This study for the first time identified CET as a key mediator of autophagy in leukemia, and the inhibition of autophagy may be a promising therapeutic strategy for leukemia.

In the present study, CET induced the apoptosis of several human leukemia cells, including HL-60, NB4, MoLT-4, Jurkat, K562 and Raji. Mitochondrial membrane permeability (MMP) is usually increased, resulting in Δψm loss, mitochondrial swelling and rupture of the mitochondrial outer membrane in drug-induced cell apoptosis [22]. Here, CET could cause the decrease of mitochondrial membrane potential and trigger mitochondria-dependent apoptosis. Previous study has revealed that Bcl-2 and Bak belong to the Bcl-2 family proteins, which regulate the mitochondria-mediated apoptosis pathway [23]. Bcl-2 has an anti-apoptotic function and maintains mitochondrial membrane integrity [24]. Bak, as a pro-apoptotic protein, accumulates at the mitochondrial outer membrane and forms an oligomer, resulting in mitochondrial membrane permeabilization [25,26]. The permeability of the mitochondrial membrane increases and, subsequently, some pro-apoptotic factors are released from mitochondria into the cytosol, leading to the activation of the caspase cascade. Caspase activation further promotes cell death [27]. As expected, CET induced mitochondria-dependent apoptosis by downregulation of the Bcl-2 level, upregulation of the Bak level and further activation of caspase-9, caspase-3 and PARP. Meanwhile, CET also caused a moderate increase of ROS in HL-60 cells. Damaged mitochondria can result in the accumulation of ROS, which plays an important role in the apoptosis process. Excessive ROS production may also interfere with mitochondrial function. In line with this, CET-induced mitochondrial damage resulted in the accumulation of ROS and in turn, excessive ROS also interfered with mitochondrial function. The mitochondrial apoptosis pathway is a classical cellular apoptotic signal pathway. Thus, directly targeting the mitochondrial apoptotic pathway may also be an effective treatment strategy for leukemia.

RNA sequencing has been widely used to investigate the expression of different genes after treatment with drugs [28,29,30]. Based on the result of RNA-sequence analysis, signaling pathways regulated by CET could be acquired, including mitophagy, death receptor signaling and NF-κB signaling pathway. In this study, the NF-κB signaling pathway was also enriched. Previous study has revealed that upon mitochondrial damage, accumulation of mtDNA and ROS can activate the NLRP3 inflammasome, leading to pro-inflammatory responses [31]. Therefore, we speculated that CET induced an increase in ROS, and excessive ROS might lead to activation of the NF-κB inflammasome signaling pathway.

Autophagy has multiple functions on tumor development and may either lead to cell death or preserve cell survival under stress conditions. However, numerous studies focus on the pro-survival function of autophagy in leukemia cells. For instance, class III PI3K complex (Vps34) is crucial for T-cell homeostasis, and Vps34-dependent canonical autophagy could promote T-cell survival by removing damaged mitochondria through mitophagy [32]. Additionally, autophagy plays a vital role for the clearance of aberrant mitochondria in hematopoietic stem cells (HSCs). Knockout of autophagy-related genes can inhibit mitophagy and result in the accumulation of damaged mitochondrial and an increase of ROS levels, which impairs the functionality of HSCs [33]. Given these observations, the inhibition of autophagy may serve as a strategy for cancer therapy. Our results also showed that CET impaired lysosomal acidification and suppressed the autophagy flux. More experiments were carried out to demonstrate this. Additionally, further inhibition of autophagy flux could aggravate cell apoptosis induced by CET.

In cells, the accumulation of damaged mitochondria can induce autophagy. Autophagy clears the damaged mitochondria and maintains cell survival. In addition, PINK1 accumulates only in damaged mitochondria, and PINK1 is involved in the initiation of mitophagy [34]. Here, we found that CET increased the level of PINK1 in HL-60 and Jurkat cells, indicating that mitophagy initiation might be induced. It is possible that mitochondria damage stimulates initiation of mitophagy. It has been reported that 3-MA inhibits the upstream of autophagy and reduces the formation of autophagosomes, which down-regulates the levels of LC3-II and p62. Then, CET blocks the downstream of autophagy and induces LC3-II and p62 accumulation. Thus, our results showed that CET in combination with 3-MA significantly reduced the levels of LC3-II and p62 compared to that in the cells treated with CET alone (Figure S2A). Meanwhile, we performed experiments to study whether inhibition of the early stage of mitophagy attenuated apoptosis induced by CET. The results showed that 3-MA inhibited the survival of HL-60 cells but did not affect CET-induced cell death (Figure S2B). Therefore, we think that inhibition of early stage of autophagy may not interfere with CET-induced cell death. We will conduct in-depth research on this in the future.

Furthermore, compared to HL-60 and Jurkat cells, K562 cells seem to be quite resistant to CET-induced cell death. Therefore, we performed experiments to explore whether CET induced the accumulation of LC3-II and p62 in K562 cells. The K562 cells were treated with CET (0, 5, 10, 20 μM) for 24 h and the expression levels of LC3-II and p62 were measured by Western blot. The results revealed that a low concentration of CET did not induce obvious accumulation of LC3-II and p62, and a high concentration of CET induced a slight accumulation of LC3-II in K562 cells (Figure S3). Therefore, compared with HL-60 and Jurkat, CET has a weaker effect on autophagy in K562 cells.

In summary, CET has a broad spectrum of antileukemia effects. Our research provides new insights into the molecular mechanisms of CET and a new therapeutic strategy for leukemia.

## 4. Materials and Methods

### 4.1. Chemicals and Reagents

CET (C_18_H_21_NO_4_) was purchased from Baoji Herbest Bio-Tech (Baoji, Shaanxi, China) with a purity greater than 98% determined by HPLC. The structure of CET is shown in Figure 1A. Penicillin–streptomycin and trypsin were obtained from Macgene (Beijing, China). Cell Counting Kit-8 (CCK-8), MitoTracker Green, LysoTracker Red and Mitochondrial Membrane Potential Assay kits were purchased from Beyotime Institute of Biotechnology (Nanjing, Jiangsu, China). The annexin V-FITC/PI apoptosis detection kit was obtained from Solarbio Science & Technology (Beijing, China). N-Acetylcysteine (NAC), chloroquine (CQ) and bafilomycin A1 (Baf A1) were purchased from MCE (Shanghai, China). Rabbit antibodies against caspase-3, cleaved caspase-3, caspase-9, cleaved caspase-9, PARP, cleaved PARP, Bcl-2, Bak, LC3B, p62, GAPDH and secondary antibodies were obtained from Cell Signaling Technology (Beverly, MA, USA). Easy II Protein Quantitative (BCA) and EasyPure Genomic DNA kits were purchased from TransGen Biotech (Beijing, China).

### 4.2. Cell Culture

Human leukemia NB4 cells (source: female) were a gift from Prof. Chen Guo-Qiang (Shanghai Jiao Tong University School of Medicine, Shanghai). Jurkat, HL-60, K562, Raji and MOLT-4 cells were purchased from Cell Resource Center, Peking Union Medical College and checked to be free of mycoplasma contamination by PCR. The species origin of cells was confirmed with PCR and the identity was authenticated with STR profiling (FBI, CODIS). All cells were cultured in RPMI 1640 medium (Gibco, Life technologies, Grand Island, NY, USA), containing 10% fetal bovine serum (Gibco, Thermo Fisher Scientific, Waltham, MA, USA), 100 mg/mL penicillin, and 100 mg/mL streptomycin at 37 °C in 5% carbon dioxide.

### 4.3. Cell Viability Assay

Cell viability was assessed by a CCK-8 assay. In brief, cells were seeded in 96-well plates with a density of 10,000 cells/well. Then, different concentrations of CET (1–100 μM) were added immediately, and cells were treated with CET for 24 h. After treatment, 10 μL CCK-8 solution was added to each well and further incubated for 4 h at 37 °C. The absorbance was read at 450 nm with a microplate reader. The cell death rate was calculated using the following Equation:

Cell death (%) = (OD_control group_ − OD_experimental group_)/(OD_control group_ − OD_blank group_) × 100% (1)

### 4.4. Mitochondrial Membrane Potential

Mitochondrial depolarization was detected by a mitochondrial membrane potential assay kit. HL-60 and Jurkat cells were seeded in six-well plates and treated with CET (0, 5, 10, 20 μM) for 24 h. Then, cells were stained with JC-1 for 20 min at 37 °C, centrifuged for 3 min at 4 °C and then washed three times with the JC-1 buffer solution. Images were captured with fluorescence microscope (Olympus, Tokyo, Japan). The red (polymeric) fluorescence was visualized under excitation wavelength 520 nm and emission wavelength 590 nm. The green (monomeric) fluorescence was visualized under excitation wavelength 490 nm and emission wavelength 530 nm.

### 4.5. Measurement of ROS

In order to measure intracellular ROS levels, HL-60 cells were pretreated with CET (0, 5, 10, 20 μM) for 24 h. 2′7′-Dichlorofluorescein diacetate (DCFH-DA, Beyotime) was diluted with serum-free medium at a ratio of 1:1000, and the final concentration was 10 μM. After the cells were collected, they were suspended in diluted DCFH-DA at a cell concentration of 1 million/mL and incubated in a cell incubator at 37 °C for 20 min. Then, the cells were washed three times with serum-free medium to completely remove the DCFH-DA, which did not enter the cells. The images were taken by a fluorescence microscope (Olympus, Tokyo, Japan).

### 4.6. Western Blot Analysis

Cells were lysed using RIPA buffer (1×) supplemented with cocktail protein inhibitors for 30 min. The concentrations of proteins were determined using Easy II Protein Quantitative Kit (TransGen Biotech, Beijing, China), respectively. Equal amounts of proteins were separated by 8-12% SDS-PAGE and transferred to polyvinylidene fluoride membranes (Millipore). Membranes were blocked in non-fat milk and probed with primary antibodies (1:1000) overnight at 4 °C. Then, the membranes were washed and incubated with the appropriate horseradish-peroxidase-coupled secondary antibody for 1 h at room temperature. Finally, protein bands were detected with a SuperSignal West Pico Chemiluminescent Substrate kit (Thermo Fisher Scientific) and acquired by Tanon 5200 Imaging System (Tanon, Shanghai, Beijing).

### 4.7. RNA Sequencing Assay and Bioinformatics Analysis

After treatment with 20 μM CET for 12 h, HL-60 cells were harvested for transcriptome sequencing. The RNA-seq assay was performed by Novogene (Beijing, China). First, total RNA was extracted by the TRIzol reagent. RNA integrity was assessed by the RNA Nano 6000 Assay Kit of the Bioanalyzer 2100 system (Agilent Technologies, CA, USA). Then, mRNA was purified with poly-T oligo-attached magnetic beads. cDNA libraries were constructed according to the manufacturer’s recommendations and library quality was assessed on the Agilent Bioanalyzer 2100 system. Differential expression analysis of two groups was carried out by DESeq R package (1.18.0). Genes with log2 (fold change) >1 were assigned as differentially expressed. The Gene Ontology (GO) enrichment analysis was performed using the Database for Annotation, Visualization and Integrated Discovery (DAVID1). The functions of differentially expressed genes were analyzed on ClueGO 2.2.5 within Cytoscape software 3.0.

### 4.8. Mitochondrial DNA (mtDNA) Analysis

HL-60 cells were treated with CET (0, 5, 10, 20 μM) for 24 h. Then, cells were harvested and the genomic DNA was extracted using EasyPure Genomic DNA Kit (TransGen Biotech, Beijing, China). Real-time quantitative PCR (qPCR) was carried out according to the protocol of Hieff^®^ qPCR SYBR Green Master Mix on a Stratagene Mx3005P (Agilent technologies, Santa Clara, CA, USA). The gene B2m-2 was used as an endogenous control for qPCR. The gene-specific primer sequences used for PCR were hMito3 forward, 5′-CATTTCCACACAGACATCA-3′ and hMito3 reverse, 5′-TGGTTAGGCTGGTGTTAGGG-3′; B2m-2 forward, 5′-GCTGGGTAGCTCTAAACAATGTATTCA-3′ and B2m-2 reverse, 5′-CCATGTACTAACAAATGTCTAAAATGGT-3′. The gene expression values were calculated by the 2^−^^△△*C*t^ method. At least three technical repetitions were conducted in this study.

### 4.9. Flow Cytometry Analysis

Apoptosis was detected with an annexin V-FITC/PI detection kit. After exposure to CET (0, 5, 10, 20 μM) for 24 h, HL-60 cells were harvested and washed twice with ice-cold PBS. Then the cells were suspended in 500 μL binding buffer and incubated with 5 μL annexin V-FITC in the dark for 10 min. Next, after staining with 5 μL PI solution for 5 min, cells were analyzed by flow cytometry. Flow cytometry analysis were performed using a Kaluza Analysis 2.1 (Kaluza software, Fullerton, CA, USA).

### 4.10. Transmission Electron Microscope (TEM) Assay

HL-60 cells were seeded in six-well plates and treated with CET (0, 10, 20 μM) for 24 h. The collected cells were washed with ice-cold PBS, centrifuged for 5 min and fixed in 4% (*w*/*v*) phosphate buffered glutaraldehyde overnight at 4 °C. Then, the fixed cells were washed three times with PBS, followed by post-fixation with 1% OsO_4_ for 2 h at 4 °C. Subsequently, cells were thoroughly dehydrated with increasing ethanol solutions, including 50%, 70%, 80%, 90% and 100% for 15 min. Finally, specimens were embedded in Epon 812, and cut into ultra- or semi-thin sections, which were observed with a JEOL 100CX electron microscope (HT7700-SS; Hitachi, Tokyo, Japan).

### 4.11. Statistical Analysis

All statistical analyses were performed by GraphPad Prism 8.0 software. The difference between two groups was analyzed using Student’s *t*-test. * *p* < 0.05 and ** *p* < 0.01 were considered as statistically significant. All data were represented as mean ± SEM. All experiments were carried out at least in triplicate.

## Data Availability

Data sharing not applicable.

## Supplementary Materials

Figure S1: The expression levels of Bcl2, Bak and LC3-II were determined by Western blot. HL-60 cells were treated with 20 μM CET for indicated time. Data are presented as the mean ± SD. **p* < 0.05 and ***p* < 0.01 versus control group, Figure S2: (A) In the absence or existence of 3-MA (2 mM), HL-60 cells were treated with CET (10 µM) for 24 h. LC3-II and p62 was measured by Western blotting. (B) Cell viability was determined by the CCK-8 assay. ***p* < 0.01, Figure S3: The expression levels of LC3-II and p62 were determined by Western blot. K562 cells were treated with CET treatment (5, 10, 20 µM) for 24 h.

## References

[1] Heendeniya S.N., Keerthirathna L.R., Manawadu C.K., Dissanayake I.H., Ali R., Mashhour A., Alzahrani H., Godakumbura P., Boudjelal M., Peiris D.C. (2020). Therapeutic efficacy of Nyctanthes arbor-tristis flowers to inhibit proliferation of acute and chronic primary human leukemia cells, with adipocyte differentiation and in silico analysis of interactions between survivin protein and selected secondary metabolites. Biomolecules.

[2] Djunic I., Virijevic M., Djurasinovic V., Novkovic A., Colovic N., Kraguljac-Kurtovic N., Vidovic A., Suvajdzic-Vukovic N., Tomin D. (2012). Prognostic significance of CD56 antigen expression in patients with acute myeloid leukemia. Med. Oncol..

[3] Kantarjian H.M., Keating M.J., Freireich E.J. (2018). Toward the potential cure of leukemias in the next decade. Cancer.

[4] Scarfò L., Ferreri A.J., Ghia P. (2016). Chronic lymphocytic leukaemia. Crit. Rev. Oncol. Hematol..

[5] Chung S.J., Nagaraju G.P., Nagalingam A., Muniraj N., Kuppusamy P., Walker A., Woo J., Győrffy B., Gabrielson E., Saxena N.K. (2017). ADIPOQ/adiponectin induces cytotoxic autophagy in breast cancer cells through STK11/LKB1-mediated activation of the AMPK-ULK1 axis. Autophagy.

[6] Djavaheri-Mergny M., Giuriato S., Tschan M.P., Humbert M. (2019). Therapeutic modulation of autophagy in leukaemia and lymphoma. Cells.

[7] Liu Q., Chen L., Atkinson J.M., Claxton D.F., Wang H.G. (2016). Atg5-dependent autophagy contributes to the development of acute myeloid leukemia in an MLL-AF9-driven mouse model. Cell Death Dis..

[8] Sumitomo Y., Koya J., Nakazaki K., Kataoka K., Tsuruta-Kishino T., Morita K., Sato T., Kurokawa M. (2016). Cytoprotective autophagy maintains leukemia-initiating cells in murine myeloid leukemia. Blood.

[9] Kovaleva V., Mora R., Park Y.J., Plass C., Chiramel A.I., Bartenschlager R., Döhner H., Stilgenbauer S., Pscherer A., Lichter P. (2012). miRNA-130a targets ATG2B and DICER1 to inhibit autophagy and trigger killing of chronic lymphocytic leukemia cells. Cancer Lett..

[10] White E. (2012). Deconvoluting the context-dependent role for autophagy in cancer. Nat. Rev. Cancer.

[11] Wang Z.H., Zhu S.C., Zhang G.S., Liu S.F. (2015). Inhibition of autophagy enhances the anticancer activity of bortezomib in B-cell acute lymphoblastic leukemia cells. Am. J. Cancer Res..

[12] Yuan N., Lin S., Zhang S.P., Lin W.W., Cao Y., Xu F., Fang Y.X., Wang Z., Zhang H., Li X. (2015). Bafilomycin A1 targets both autophagy and apoptosis pathways in pediatric B-cell acute lymphoblas-tic leukemia. Haematologica.

[13] Lu S., Wang J. (2014). Homoharringtonine and omacetaxine for myeloid hematological malignancies. J. Hematol. Oncol..

[14] Lai Z.Z., Ho Y.J., Lu J.W. (2020). Cephalotaxine inhibits Zika infection by impeding viral replication and stability. Biochem. Biophys. Res. Commun..

[15] Romero M.R., Serrano M.A., Efferth T., Alvarez M., Marin J.J. (2007). Effect of cantharidin, cephalotaxine and homoharringtonine on “in vitro” models of hepatitis B virus (HBV) and bovine viral diarrhoea virus (BVDV) replication. Planta Med..

[16] Efferth T., Sauerbrey A., Halatsch M.E., Ross D.D., Gebhart E. (2003). Molecular modes of action of cephalotaxine and homoharringtonine from the coniferous tree *Cephalotaxus hainanensis* in human tumor cell lines. Naunyn Schmiedebergs Arch. Pharmacol..

[17] He L., Gu K. (2018). Tanshinone IIA regulates colorectal cancer apoptosis via attenuation of Parkin-mediated mitophagy by suppressing AMPK/Skp2 pathways. Mol. Med. Rep..

[18] Wei R., Cao J., Yao S. (2018). Matrine promotes liver cancer cell apoptosis by inhibiting mitophagy and PINK1/Parkin pathways. Cell Stress Chaperones..

[19] León-Aparicio D., Salvador C., Aparicio-Trejo O.E., Briones-Herrera A., Pedraza-Chaverri J., Vaca L., Sampieri A., Padilla-Flores T., López-González Z., León-Contreras J.C. (2019). Novel potassium channels in kidney mitochondria: The hyperpolarization-activated and cyclic nucleotide-gated HCN channels. Int. J. Mol. Sci..

[20] Li H., Pan Y., Wu H., Yu S., Wang J., Zheng J., Wang C., Li J., Jiang J. (2020). Inhibition of excessive mitophagy by N-acetyl-L-tryptophan confers hepatoprotection against Ischemia-Reperfusion injury in rats. PeerJ.

[21] Messling S., Matthias J., Xiong Q., Fischer S., Eichinger L. (2017). The two Dictyostelium discoideum autophagy 8 proteins have distinct autophagic functions. Eur. J. Cell Biol..

[22] Tsujimoto Y., Shimizu S. (2007). Role of the mitochondrial membrane permeability transition in cell death. Apoptosis.

[23] Desagher S., Martinou J.C. (2000). Mitochondria as the central control point of apoptosis. Trends Cell Biol..

[24] Chipuk J.E., Green D.R. (2008). How do BCL-2 proteins induce mitochondrial outer membrane permeabilization?. Trends Cell Biol..

[25] Danial N.N. (2007). BCL-2 family proteins: Critical checkpoints of apoptotic cell death. Clin. Cancer Res..

[26] Estaquier J., Vallette F., Vayssiere J.L., Mignotte B. (2012). The mitochondrial pathways of apoptosis. Adv. Exp. Med. Biol..

[27] Tait S.W., Green D.R. (2010). Mitochondria and cell death: Outer membrane permeabilization and beyond. Nat. Rev. Mol. Cell Biol..

[28] Hadwen J., Schock S., Mears A., Yang R., Charron P., Zhang L., Xi H.S., MacKenzie A. (2018). Transcriptomic RNAseq drug screen in cerebrocortical cultures: Toward novel neurogenetic disease therapies. Hum. Mol. Genet..

[29] Wang Z., Gerstein M., Snyder M. (2009). RNA-Seq: A revolutionary tool for transcriptomics. Nat. Rev. Genet..

[30] Zhang H., Chen Q., Dahan A., Xue J., Wei L., Tan W., Zhang G. (2019). Transcriptomic analyses reveal the molecular mechanisms of schisandrin B alleviates CCl4-induced liver fibrosis in rats by RNA-sequencing. Chem. Biol. Interact..

[31] Lin Q., Li S., Jiang N., Shao X., Zhang M., Jin H., Zhang Z., Shen J., Zhou Y., Zhou W. (2019). PINK1-parkin pathway of mitophagy protects against contrast-induced acute kidney injury via decreasing mitochondrial ROS and NLRP3 inflammasome activation. Redox Biol..

[32] Willinger T., Flavell R.A. (2012). Canonical autophagy dependent on the class III phosphoinositide-3 kinase Vps34 is required for naive T-cell homeostasis. Proc. Natl. Acad. Sci. USA.

[33] Ho T.T., Warr M.R., Adelman E.R., Lansinger O., Flach J., Verovskaya E.V., Figueroa M.E., Passegué E. (2017). Autophagy maintains the metabolism and function of young and old stem cells. Nature.

[34] Kubli D.A., Gustafsson Å.B. (2012). Mitochondria and mitophagy: The yin and yang of cell death control. Circ. Res..

