# Supplementary material for: Cephalotaxine Inhibits the Survival of Leukemia Cells by Activating Mitochondrial Apoptosis Pathway and Inhibiting Autophagy Flow
Source: Molecules. 2021 May 18;26(10):2996. doi: 10.3390/molecules26102996 (PMC8158396; doi:10.3390/molecules26102996)
Supplement: Supplementary file 1 [file molecules-26-02996-s001.zip › molecules-1213673-supplementary.pdf]

Supplementary Materials

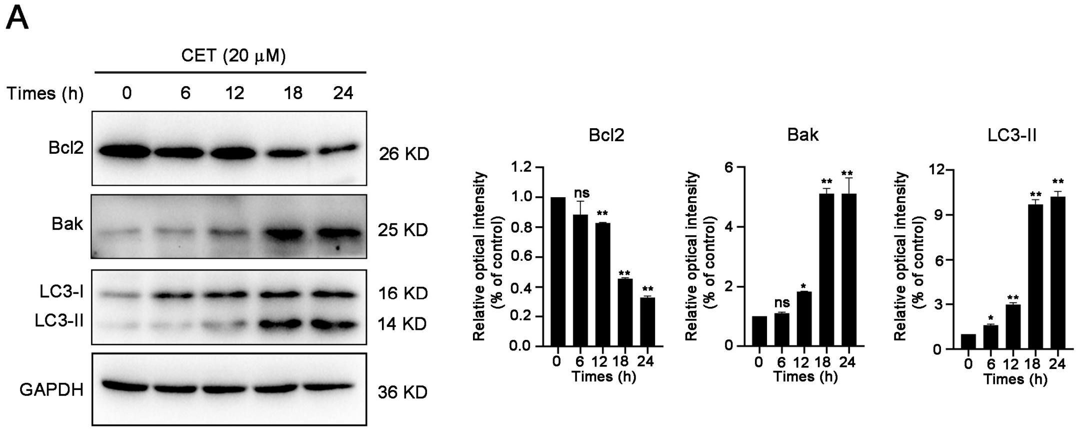

Figure 1. The expression levels of Bcl2, Bak and LC3-II were determined by Western blot. HL-60 cells were treated with 20 μM CET for indicated time. Data are presented as the mean ± SD. **p* < 0.05 and ***p* < 0.01 versus control group.

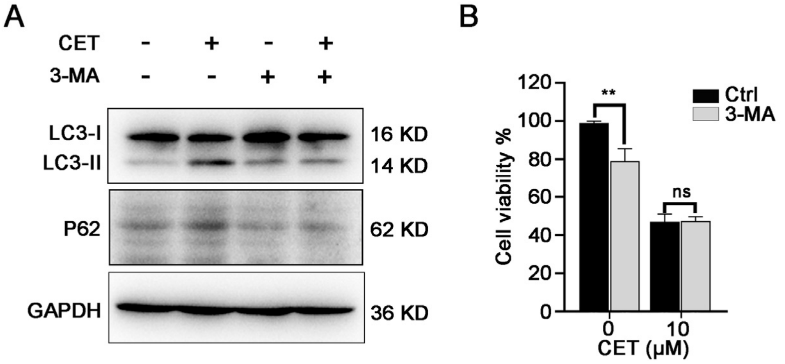

Figure 2. (**A**) In the absence or existence of 3-MA (2 mM), HL-60 cells were treated with CET (10 μM) for 24 h. LC3-II and p62 was measured by Western blotting. (**B**) Cell viability was determined by the CCK-8 assay. ***p* < 0.01.

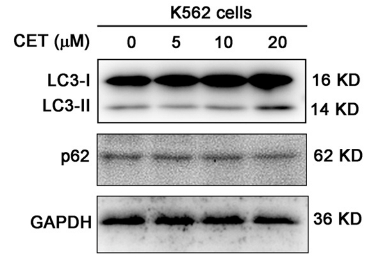

Figure 3. The expression levels of LC3-II and p62 were determined by Western blot. K562 cells were treated with CET treatment (5, 10, 20 μM) for 24 h.

Original Images for Western Blot's

Figure 2C

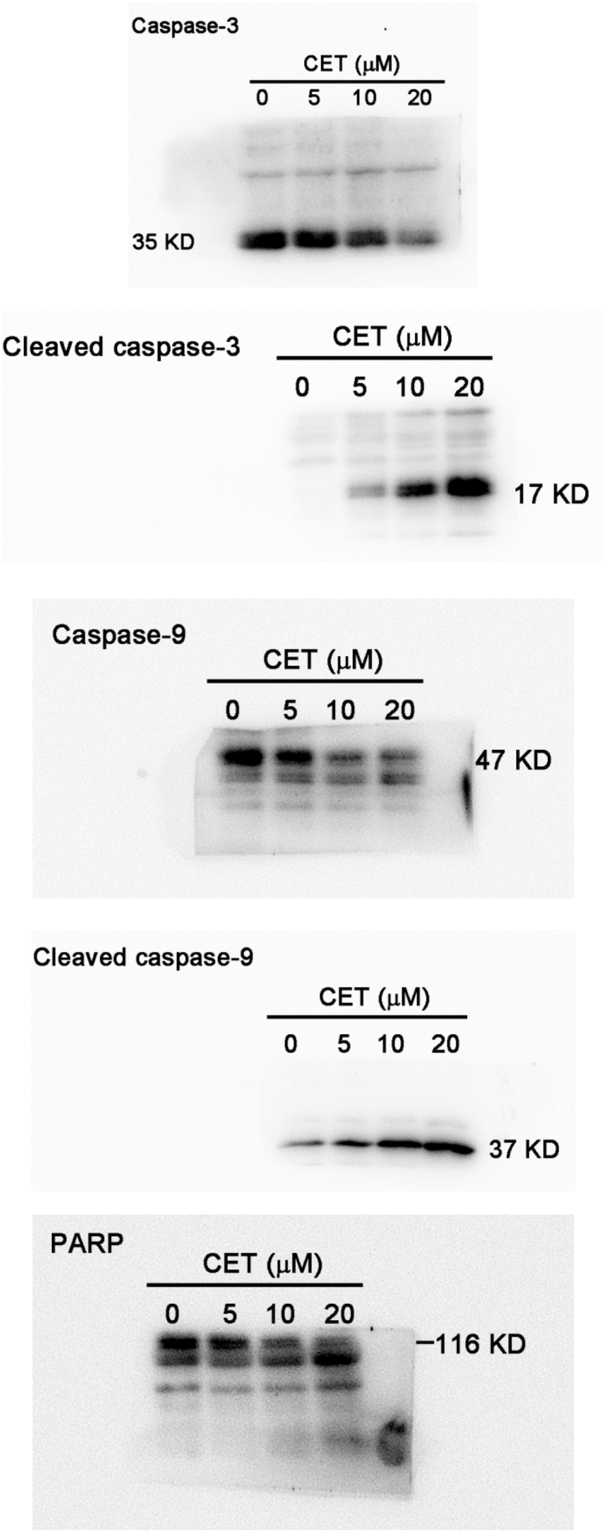

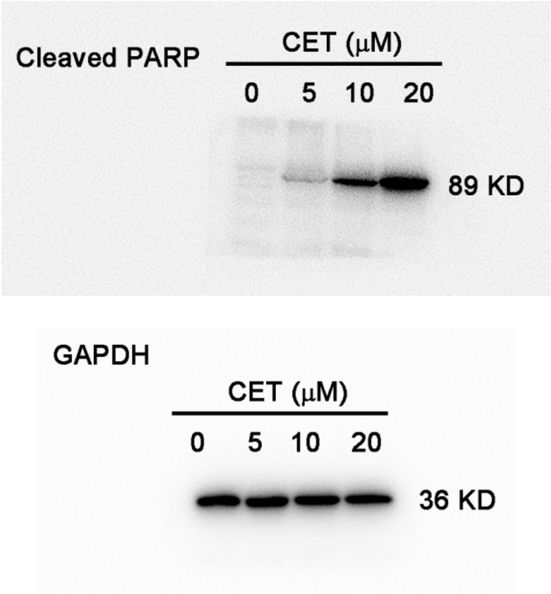
Cleaved PARP
CET (μM)
0 5 10 20
89 KD
GAPDH
CET (μM)
0 5 10 20
36 KD

Figure 2D

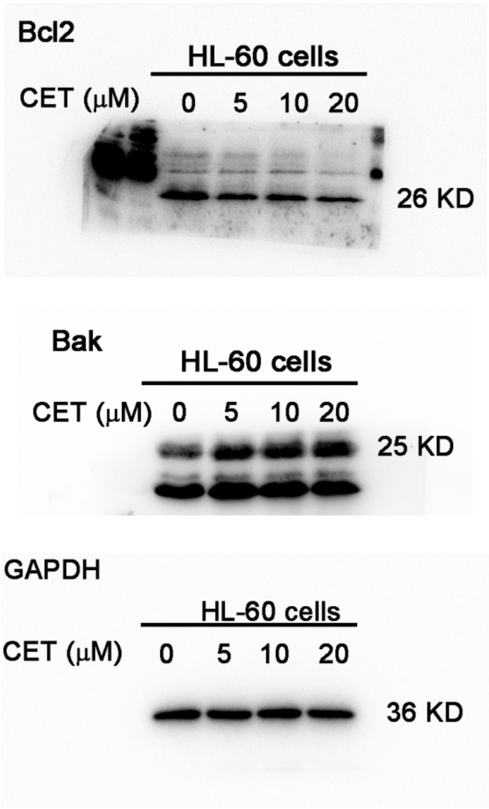
Bcl2
HL-60 cells
CET (μM) 0 5 10 20
26 KD
Bak
HL-60 cells
CET (μM) 0 5 10 20
25 KD
GAPDH
HL-60 cells
CET (μM) 0 5 10 20
36 KD

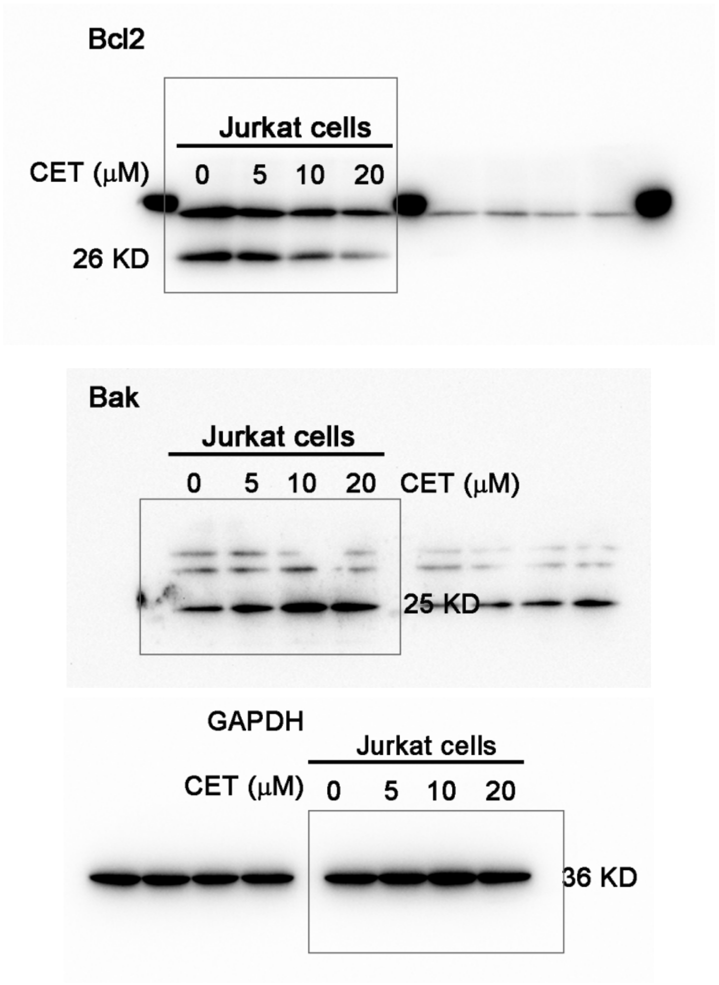
Bcl2
Jurkat cells
CET (μM) 0 5 10 20
26 KD
Bak
Jurkat cells
0 5 10 20 CET (μM)
25 KD
GAPDH
Jurkat cells
CET (μM) 0 5 10 20
36 KD

Figure 4E

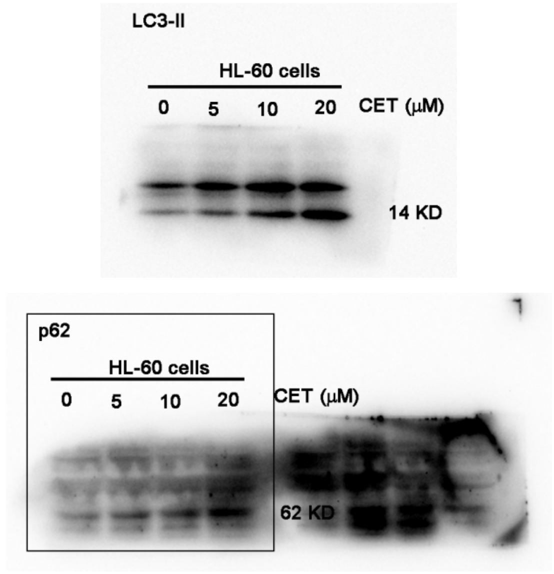
LC3-II
HL-60 cells
0 5 10 20 CET (μM)
14 KD
p62
HL-60 cells
0 5 10 20
CET (μM)
62 KD

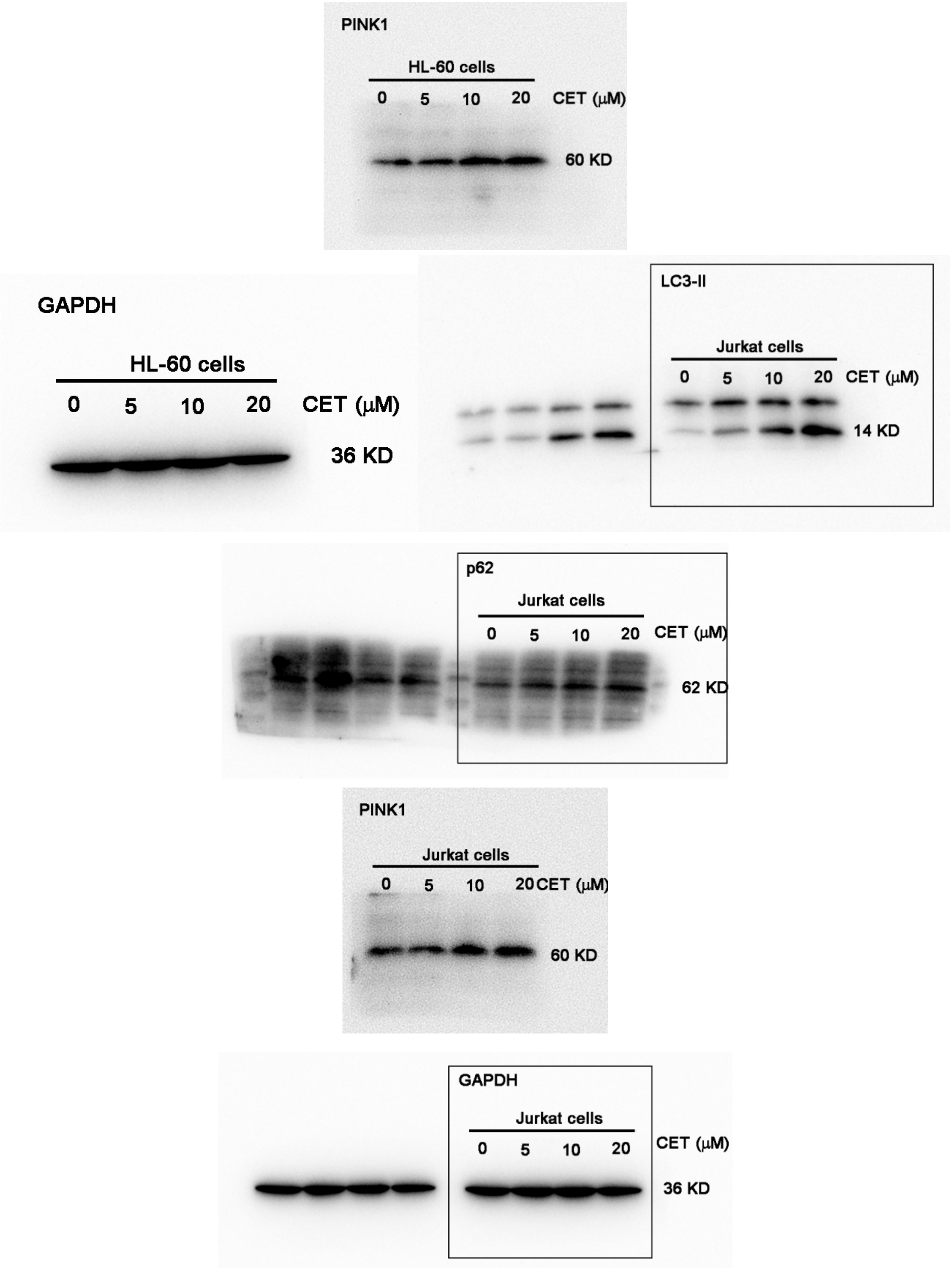
PINK1
HL-60 cells
0 5 10 20 CET (μM)
60 KD
GAPDH
HL-60 cells
0 5 10 20 CET (μM)
36 KD
LC3-II
Jurkat cells
0 5 10 20 CET (μM)
14 KD
p62
Jurkat cells
0 5 10 20 CET (μM)
62 KD
PINK1
Jurkat cells
0 5 10 20 CET (μM)
60 KD
GAPDH
Jurkat cells
0 5 10 20 CET (μM)
36 KD

Figure 5A

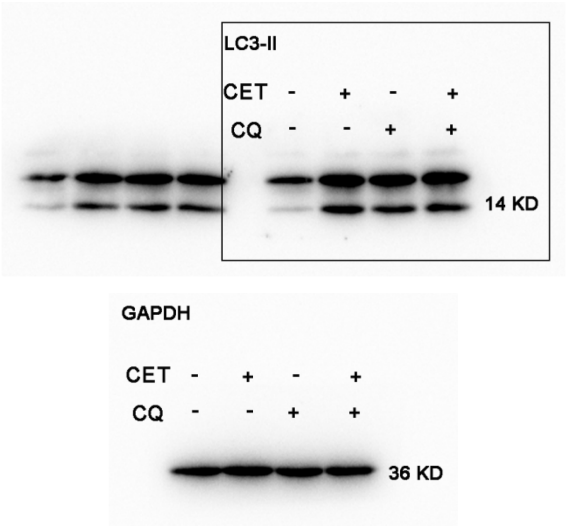

Figure 5B

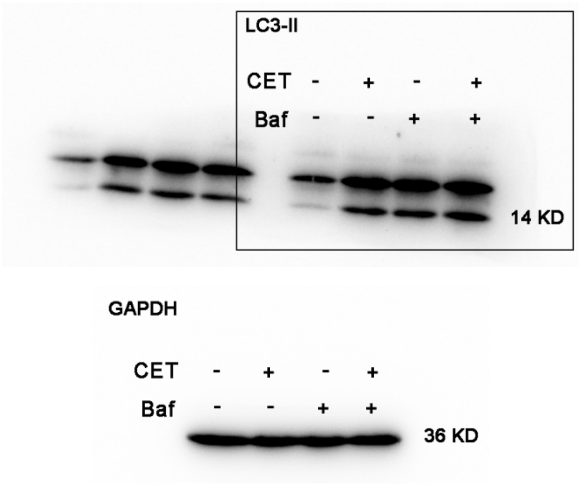

Figure 5C

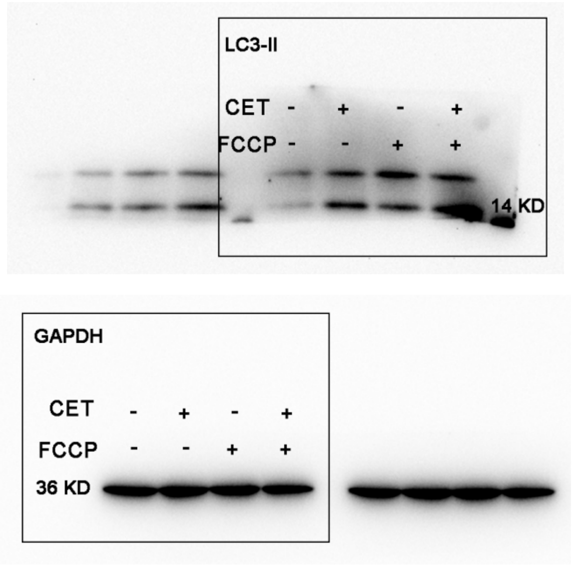

Supplementary materials

Figure S1

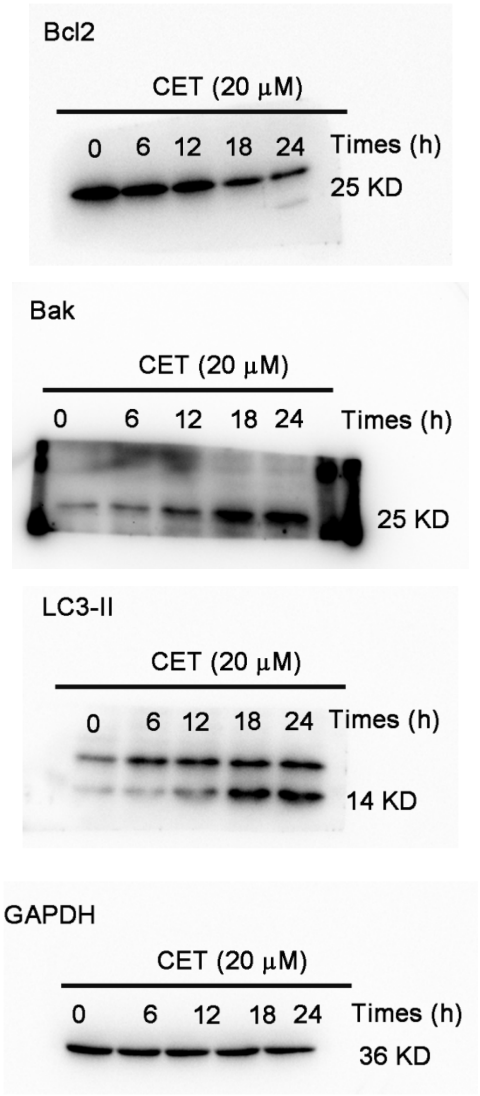

Figure S2

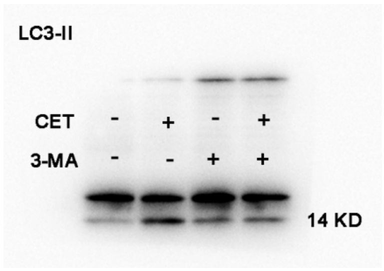

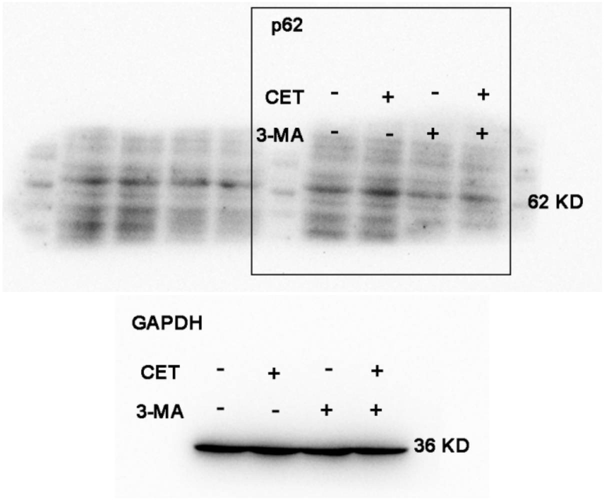
p62
CET - + - +
3-MA - - + +
62 KD
GAPDH
CET - + - +
3-MA - - + +
36 KD

Figure S3

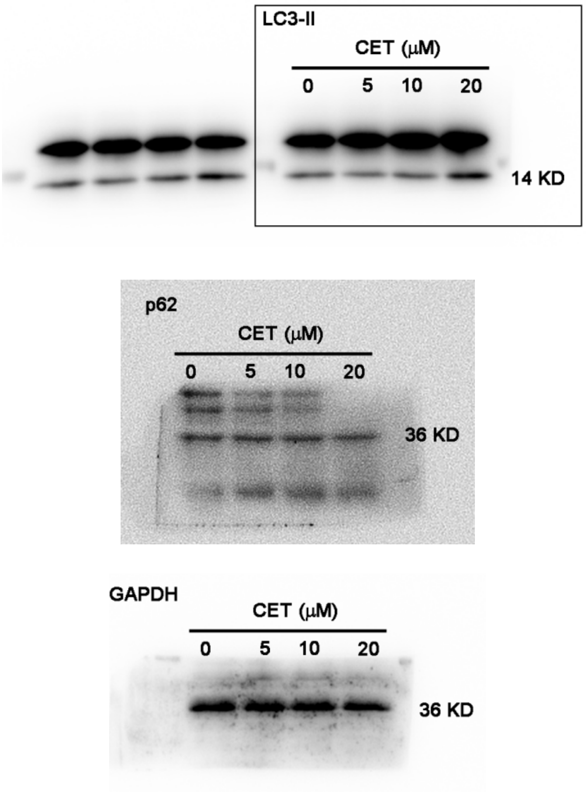
LC3-II
CET (μM)
0 5 10 20
14 KD
p62
CET (μM)
0 5 10 20
36 KD
GAPDH
CET (μM)
0 5 10 20
36 KD
